# Expanded conventional first trimester screening

**DOI:** 10.1002/pd.5090

**Published:** 2017-07-18

**Authors:** Jonathan B. Carmichael, Hsiao‐Pin Liu, David Janik, Terrence W. Hallahan, Kypros H. Nicolaides, David A. Krantz

**Affiliations:** ^1^ Eurofins NTD, LLC Melville NY USA; ^2^ Harris Birthright Research Centre for Fetal Medicine King's College Hospital London UK

## Abstract

**Objective:**

The study aims to determine the performance of a five (5) serum marker plus ultrasound screening protocol for T21, T18 and T13.

**Method:**

Specimens from 331 unaffected, 34 T21, 19 T18 and 8 T13 cases were analyzed for free Beta human chorionic gonadotropin, pregnancy‐associated plasma protein A, alpha‐fetoprotein, placental growth factor and dimeric inhibin A. Gaussian distributions of multiples of the median values were used to estimate modeled false positive and detection rates (DR).

**Results:**

For T21, at a 1/300 risk cut‐off, DR of screening with all five serum markers along with nuchal translucency and nasal bone was 98% at a 1.2% false positive rate (FPR). Using a 1/1000 cut‐off, the DR was 99% with a 2.6% FPR. For T18/13 with free Beta human chorionic gonadotropin, pregnancy‐associated plasma protein A, placental growth factor and nuchal translucency at a 1/150 cut‐off, DR was 95% at a 0.5% FPR while at a 1/500 risk cut‐off, DR was 97% at a 1.2% FPR.

**Conclusion:**

An expanded conventional screening test can achieve very high DRs with low FPRs. Such screening fits well with proposed contingency protocols utilizing cell‐free DNA as a secondary or reflex but also provides the advantages of identification of pregnancies at risk for other adverse outcomes such as early‐onset preeclampsia. © 2017 Eurofins NTD, LLC. *Prenatal Diagnosis* published by John Wiley & Sons, Ltd.

## Abstract 2

**What's already known about this topic?**

- Conventional Down syndrome screening with ultrasound markers and free Beta hCG and PAPP‐A has been successfully utilized for nearly 20 years to screen for trisomies 21, 18 and 13.
- Cell‐free DNA screening has much higher detection and lower false positive rates but is expensive.

**What does this study add?**

- An expanded conventional screen with nuchal translucency and nasal bone that includes additional serum markers AFP, placental growth factor and dimeric inhibin A can detect 98% of trisomy 21 and 95% of trisomy 18/13 cases at a false positive rate of 1.2% and 0.5%, respectively.
- Offering cell‐free DNA testing to those patients found at increased risk with the expanded screen maintains the detection efficiency but brings the invasive testing rate to an exceedingly low level.

## Introduction

Aneuploidy screening has continued to grow and evolve since its inception in the 1980s. Initially, conventional Down syndrome screening took place in the second trimester using alpha‐fetoprotein (AFP) only and would eventually expand to include total or free Beta human chorionic gonadotropin (hCG), unconjugated estriol and dimeric inhibin A (DIA).^1^, ^2^, ^3^, ^4^, ^5^ By the late 1990s, an alternative screen in the first trimester using ultrasound (e.g. nuchal translucency, nasal bone) and biochemical markers, free Beta hCG and pregnancy‐associated plasma protein A (PAPP‐A) was introduced.^6^, ^7^ More recently, AFP, Inhibin and placental growth factor (PlGF) have been incorporated into the first trimester screen.^8^, ^9^, ^10^, ^11^

Recently, a new screening technology using cell‐free fetal DNA from maternal circulation has been introduced.^12^, ^13^, ^14^, ^15^ This screening is characterized by high detection rates with low false positive rates but has an associated failure rate of 1–5%.^16^ The failure rate coupled with the significant cost of the screen has hampered universal adoption of cell‐free fetal DNA technology. To that end, several studies have suggested implementing a contingent approach in which conventional screening would be used to identify high‐risk individuals who would then be offered cell‐free fetal DNA testing.^17^, ^18^, ^19^, ^20^ This tiered approach to screening enables cost‐effectiveness, high detection rates, reduction in invasive procedures and a broader evaluation of the health of the pregnancy. Although, historically, prenatal screening has focused on identifying fetal abnormalities, recent focus has been on maternal complications in pregnancy such as preeclampsia, gestational diabetes and preterm birth. Preeclampsia is the leading cause of pregnancy‐related morbidity and mortality affecting 5% of pregnancies worldwide.^21^ Several different combinations of first trimester biochemical and biophysical markers have been suggested as screening tools. The most common biophysical markers are mean arterial pressure and uterine artery Doppler pulsatility index, while the most promising biochemical markers include PAPP‐A, PlGF and AFP.^22^, ^23^, ^24^ Recent meta‐analyses have indicated that treatment with low‐dose aspirin prior to 16 weeks significantly reduces the incidence of preeclampsia, especially the severe and early onset form of the disease.^25^, ^26^ Additionally, Park *et al*. found in a retrospective analysis of two consecutive cohorts of women screened for early preeclampsia that there was a 90% reduction in early‐onset preeclampsia in the cohort in which aspirin was offered to screen positive patients compared with the cohort in which no intervention occurred.^27^ As a result, a first trimester screen that identified patients at high risk for preeclampsia combined with follow‐up treatment of low‐dose aspirin could result in a significant improvement in patient care. The ability to perform conventional first‐trimester aneuploidy screening with greater detection rates than currently available while simultaneously screening for preeclampsia would have significant advantages.

We sought to evaluate the potential of a five biomarker aneuploidy screen including free Beta hCG, PAPP‐A, AFP, PlGF and DIA in both a conventional and contingent manner.

## Materials and Methods

### Study population

This was a case–control study drawn from a large prospective observational study for early prediction of pregnancy complications in women attending for their routine first hospital visit in pregnancy at King's College Hospital, London, UK. At this visit, which was held at 11 + 0 to 13 + 6 weeks' gestation, maternal characteristics and medical history were recorded and an ultrasound scan was performed to, first, confirm gestational age from the measurement of the fetal crown‐rump length,^28^ second, diagnose any major fetal abnormalities^29^ and, third, screen for chromosomal abnormalities based on fetal nuchal translucency thickness and maternal serum pregnancy‐associated plasma protein‐A and free ß‐hCG.^30^, ^31^ In addition, all fetuses were evaluated for the absence or presence of nasal bone. Women attending for this visit were invited to participate in a study on the prediction of pregnancy complications, and from those who provided informed written consent, serum samples were stored at −80°C for subsequent biochemical analysis. The study was approved by the National Research Ethics Committee.

Data on pregnancy outcome were obtained from the maternity computerized records or the general medical practitioners of the women. The cases of aneuploidies were selected at random from the stored samples, and each case was matched to five controls that were sampled on the same or next day. The controls were normal pregnancies without pregnancy complications resulting in live birth after 37 weeks' gestation of phenotypically normal neonates with birth weight between the 5th and 95th percentiles for gestational age.^32^ All affected pregnancies were confirmed by cytogenetic testing. None of the samples were previously thawed and refrozen. There were 27 specimens excluded due to insufficient volume and 1 specimen excluded for late gestational age resulting in a study data set of 331 unaffected, 34 T21, 19 T18 and 8 T13 cases.

### Patient characteristics

Table 1 provides a breakdown of the demographic variables for unaffected and trisomies 21, 18 and 13. There was no significant difference between the groups in terms of weight, smoking, artificial reproduction techniques and ethnicity. The trisomy 21, 18 and 13 cases were significantly older than the unaffected cases, consistent with the known association of maternal age with incidence of trisomy (Table 1).^33^, ^34^ The estimated gestational age of the trisomy 21 cases was approximately 1 day less while for trisomy 18 and 13 cases, the gestational age was 4 days less than in unaffected cases (Table 1). This observation may be due to the fact that gestational age was based on crown‐rump length (CRL), and trisomy‐affected fetuses are likely to have first trimester intrauterine growth restriction, especially in cases of trisomy 18 and 13.^35^

**Table 1:** Demographic data

	Unaffected	T21	T18	T13	
N	331	34	19	8	*P* value
Maternal age – Avg(SD)	30.9 (5.75)	36.9 (6.29)	39.0 (3.51)	33.7 (7.10)	<0.0001
Gestational days – Avg (SD)	89.7(3.22)	88.4(3.81)	85.3(3.90)	85.1(1.55)	<0.0001
Weight	150.4 (31.5)	147.4 (27.1)	158.4 (32.1)	154.5 (17.9)	0.44
Smokers	24 (7.25%)	6 (17.6%)	0 (0.0%)	1 (12.5%)	0.08
ART	12 (3.6%)	2 (5.9%)	3 (15.8%)	0 (0.0%)	0.10
Ethnicity					0.24
Caucasian	200 (60.4%)	29 (85.3%)	14 (73.7%)	7 (87.5%)	
Afro Caribbean	88 (26.6%)	2 (5.9%)	5 (26.3%)	1 (12.5%)	
East Asian	9(2.8%)	1(2.9%)	0	0	
South Asian	19(5.7%)	2(5.9%)	0	0	
Mixed	15(4.5%)	0	0	0	

### Assay methodology

All assays used were time resolved fluorometry sandwich immunoassays performed using PerkinElmer AutoDELFIA instruments. All assays were previously approved for clinical use by the New York State Department of Health for second trimester Down syndrome (free beta hCG and DIA) or first trimester preeclamspsia (AFP, PAPP‐A, PlGF) serum screening. All testing was performed at Eurofins NTD, LLC. (Melville, NY, USA). Intra‐assay and inter‐assay variation was 4.7% and 4.7% for PlGF, 1.9% and 1.6% for AFP, 7.9% and 3.0% for PAPP‐A, 5.0% and 3.7% for Free Beta hCG and 4.0% and 1.4% for DIA.

### Statistical analysis

Concentration levels were converted to multiples of the median (MoM) values by log‐linear regression of the observed analyte medians among Caucasian patients versus gestational age (grouped in 2‐day intervals). MoM values were then adjusted for maternal weight, and ethnicity and smoking. Weight adjustment values were determined by log–log regression of unadjusted MoMs versus maternal weight. After weight adjustment, ethnicity and smoking adjustments were determined based on the overall observed median weight‐adjusted MoM for each analyte among the various ethnic groups (Afro Caribbean, East Asian, South Asian, Mixed) and smokers. If the observed weight‐adjusted MoM was not significantly different from the baseline group (Caucasian, non‐smokers), then no adjustment was used.

Screening performance was determined based on Gaussian modeling. Parameters for log‐Gaussian distributions were calculated for unaffected, Down syndrome and a combination of trisomy 18 and 13 cases. All five serum markers were included in the Down syndrome risk assessment, while trisomy 18 and 13 risk assessment included free beta hCG, PAPP‐A and PlGF. Trisomy 18 and 13 cases were combined because of the limited number of cases with these outcomes. The affected mean MoM values for each analyte were based on the log of the median MoM. The unaffected mean was set equal to 0 for each analyte. The standard deviation for each distribution was determined by subtracting the tenth percentile from the 90th percentile on a log scale and dividing by 2.563. For correlation parameters between biochemical markers, spearman correlation coefficients were used. For the distribution parameters for nuchal translucency (NT), published data were used^36^ and the correlation between each biochemical marker and NT was set equal to 0. False positive and detection rates were determined by simulation based on the Gaussian distribution parameters. MoM values for biochemistry and nuchal translucency were simulated using the parameters of the Gaussian distributions. Simulated absent and present nasal bone results were based on published rates.^37^

A likelihood ratio for each simulated patient was determined from the height of the Gaussian distribution of affected cases to the height of the Gaussian distribution in unaffected cases based on the simulated MoM values. The likelihood ratio for absence and presence of nasal bone was calculated as previously described.^9^, ^37^ The likelihood ratio for nasal bone was multiplied by the likelihood ratio from the Gaussian distributions. Risk values were determined by multiplying the likelihood ratio by the *a priori* risk. For each maternal age, an age‐specific false positive and detection rate was determined based on the percentage of simulated results above the cut‐off risk. An overall screening false positive rate and detection rate was determined by weighting these percentages by the age distribution of live births in the USA in 2012.^38^

The invasive rate and final detection rate were determined by multiplying the screening rates by their associated rates for cfDNA testing alone. For cfDNA testing alone, the false positive rate was set to the sum of the false positive rate in called results times the call rate plus the no‐call rate while the detection rate was set to the sum of the detection rate in called results times the call rate plus the no‐call rate. For trisomy 18 and 13, the detection rate in called results was based on the weighted average of these rates in trisomy 18 and trisomy assuming that trisomy 18 was three times as common as trisomy 13. A 4% no‐call rate was assumed. Based on the data from the Gil Metaanalysis^16^ and the previous formulas, the associated false positive rates for cfDNA testing alone for trisomy 21 and trisomy 18/13 were 4.16% and 4.26%, respectively, while the associated detection rates were 99.232% and 95.176%, respectively.

## Results

Table 2 shows the median MoM and standard deviation for each analyte in the unaffected, trisomy 21 and trisomy 18/13 populations. The median MoM of all analytes was significantly different in the T21 population compared with the unaffected population. In the trisomy 18/13 population, PAPP‐A, free Beta hCG and PlGF were significantly different from unaffected while AFP and DIA were not significantly different. Table 3 provides the correlation coefficients between each pair of markers in the unaffected, trisomy 21 and trisomy 18/13 populations. All correlations were relatively small (<0.4) except for the correlation of inhibin and free Beta hCG in both the unaffected group and and trisomy 18/13 group and free Beta hCG and PAPP‐A in the trisomy 18/13 group.

**Table 2:** Median MoM and SD ln MoM distribution parameters in unaffected, T21 and T18/13 cases

	Median MoM			SD ln(MoM)		
	Unaffected *n* = 331	T21 *n* = 34	T18/13 *n* = 27	Unaffected *n* = 331	T21 *n* = 34	T18/13 *n* = 27
Free Beta hCG	1.00	2.23***	0.26***	0.5583	0.3830	0.7741
PAPP‐A	1.00	0.42***	0.17***	0.5776	0.6748	0.7182
AFP	1.00	0.81**	1.02	0.4300	0.3275	0.8807
PlGF	1.00	0.53***	0.55***	0.3940	0.4019	0.4111
DIA	1.00	1.90***	1.02	0.4169	0.4378	0.5656

AFP, alpha‐fetoprotein; DIA, dimeric inhibin A; MoM, multiples of the median; PAPP‐A, pregnancy‐associated plasma protein A; PlGF, placental growth factor; SD, standard deviation.

* *P* < 0.05.

** *P* < 0.01.

*** *P* < 0.001.

**Table 3:** MoM correlation coefficients

	Free Beta hCG	PAPP‐A	AFP	PLGF	DIA
**Unaffected**					
Free Beta hCG	1.0000	0.1912	−0.0237	0.0898	0.5324
PAPP‐A	0.1912	1.0000	0.0883	0.2509	0.2662
AFP	−0.0237	0.0883	1.0000	−0.0452	0.0374
PlGF	0.0898	0.2509	−0.0452	1.0000	0.0047
DIA	0.5324	0.2662	0.0374	0.0047	1.0000
**T21**					
Free Beta hCG	1.0000	−0.0500	−0.0656	0.0112	0.2862
PAPP‐A	−0.0500	1.0000	0.0426	0.1997	0.2223
AFP	−0.0656	0.0426	1.0000	0.1132	−0.0435
PlGF	0.0112	0.1997	0.1132	1.0000	−0.3479
DIA	0.2862	0.2223	−0.0435	−0.3479	1.0000
**T18/13**					
Free Beta hCG	1.0000	0.4628	0.0568	−0.1300	0.4057
PAPP‐A	0.4628	1.0000	−0.1819	0.1850	0.3532
AFP	0.0568	−0.1819	1.0000	−0.3309	−0.0244
PlGF	−0.1300	0.1850	−0.3309	1.0000	−0.2510
DIA	0.4057	0.3532	−0.0244	−0.2510	1.0000

AFP, alpha‐fetoprotein; DIA, dimeric inhibin A; hCG, human chorionic gonadotropin; MoM, multiples of the median; PAPP‐A, pregnancy‐associated plasma protein A; PlGF, placental growth factor; SD, standard deviation.

Table 4 shows the performance of screening protocols at fixed false positive and detection rates. At a fixed 5% false positive rate, the detection rate of Trsiomy 21 was 93%, 98% and 99% for serum markers only, serum markers plus nuchal translucency and serum markers plus nuchal translucency and nasal bone. For Trisomy 18/13 at a fixed 1% false positive rate, the detection rate was 86% and 97% for serum markers only and serum markers plus nuchal translucency. Table 5 shows the false positive and detection rates for various screening protocols at various risk cutoffs for trisomy 21 and trisomy 18/13 as well as the ultimate detection rate and invasive testing rate if all positive screening results were followed by cell‐free DNA testing. Using a standard 1/300 cut‐off risk with follow‐up by invasive testing procedure, the trisomy 21 detection rate was 92%, 95% and 98% based on a serum only, serum plus nuchal translucency and serum plus nuchal translucency plus nasal bone protocol, respectively. The corresponding false positive rate would be 4.8%, 2.3% and 1.2%, respectively. For trisomy 18/13, at a 1/150 cut‐off risk, the detection rate was 88% and 95% for serum only and serum plus nuchal translucency protocol, respectively. The corresponding false positive rate was 1.2% and 0.5%, respectively. Factoring in follow‐up with cfDNA testing prior to offering invasive testing would result in detection rates of 91.4%, 94.7% and 96.9% at corresponding invasive testing rates of 0.2%, 0.1% and 0.05% for Trisomy 21 using serum markers only, serum plus NT and serum plus NT plus nasal bone, respectively. For trisomy 18/13, the detection rates would be 83.7% for serum markers only and 90.5% for serum markers plus NT at corresponding invasive testing rates of 0.05% and 0.02%.

**Table 4:** Modeled screening performance of trisomy 21 and 18/13 screening using serum and/or ultrasound markers at various risk cut‐offs

	2% FPR		5% FPR		90% DR		95% DR	
Protocol	Cut‐off	DR	Cut‐off	DR	Cut‐off	FPR	Cut‐off	FPR
T21, Serum	97	86%	320	93%	200	3.6%	615	7.8%
T21, Serum + NT	234	95%	880	98%	55	0.6%	260	2.2%
T21, Serum + NT + NB	620	98%	2750	99%	10	0.1%	60	0.4%

	0.5% FPR		1% FPR		90% DR		95% DR	
Protocol	Cut‐off	DR	Cut‐off	DR	Cut‐off	FPR	Cut‐off	FPR
T18/13, Serum	50	82%	110	86%	240	1.8%	950	5.2%
T18/13,Serum + NT	167	95%	400	97%	20	0.1%	150	0.5%

AFP, alpha‐fetoprotein; DIA, dimeric inhibin A; DR, detection rate; FPR, false positive rate; hCG, human chorionic gonadotropin; PAPP‐A, pregnancy‐associated plasma protein A; PlGF, placental growth factor.

T21, Serum = free Beta hCG, PAPP‐A, AFP, PlGF, DIA. T18/13, Serum = free Beta hCG, PAPP‐A, PlGF. Results do not factor in cell‐free DNA testing.

If the serum markers were limited to free Beta hCG and PAPP‐A, at a 1/300 risk cut‐off, the screening detection rates would have been 86%, 92% and 96% at a corresponding false positive rate of 8.4%, 3.7% and 1.9% for serum markers only, serum plus NT and serum plus NT and nasal bone, respectively. For Trisomy 18/13, using a 1/150 risk cut‐off and limiting the serum markers to free Beta hCG and PAPP‐A, the screening detection rates would have been 86% and 94% at a corresponding false positive rate of 1.3% and 0.5% for serum markers and serum markers plus nuchal translucency.

## Discussion

Our study demonstrated the improved performance of first trimester screening when additional biochemical markers (AFP, PlGF and DIA) were added to the standard protocol and represents an alternative to universal cell‐free DNA testing either in a conventional or contingent manner. Our data are in agreement with other studies that have shown improved performance when additional serum markers are added to expanded first trimester screening.^8^, ^11^, ^39^ Inclusion of nasal bone provides improved screening performance and has the advantage of not increasing the cost of the screen.

**Table 5:** Performance of screening alone and with cell‐free DNA follow‐up testing for trisomy 21 and 18/13 screening at various risk cut‐offs

T21 risk cut‐off	**1/300**				**1/1000**				**1/2500**			
Protocol	FPR	SDR	IR	DR	FPR	SDR	IR	DR	FPR	SDR	IR	DR
T21, Serum	4.8%	92%	0.2%	91.4%	10.4%	97%	0.44%	95.7%	17.2%	98%	0.7%	97.5%
T21, Serum + NT	2.3%	95%	0.1%	94.7%	5.2%	98%	0.22%	96.9%	9.1%	99%	0.4%	98.0%
T21, Serum + NT + NB	1.2%	98%	0.05%	96.9%	2.6%	99%	0.11%	98.0%	4.7%	99%	0.2%	98.5%

T18/13 Risk Cut‐Off	1/150				1/500				1/1000			
Protocol	FPR	SDR	IR	DR	FPR	SDR	IR	DR	FPR	SDR	IR	DR
T18/13, Serum	1.2%	88%	0.05%	83.7%	3.3%	93%	0.1%	88.6%	5.0%	95%	0.2%	90.7%
T18/13,Serum + NT	0.5%	95%	0.02%	90.5%	1.2%	97%	0.05%	92.3%	1.9%	98%	0.08%	93.1%

AFP, alpha‐fetoprotein; DIA, dimeric inhibin A; DR, detection rate after screening and follow up with cfDNA testing; FPR, false positive rate of screening at the given risk cutoff; hCG, human chorionic gonadotropin; IR, invasive testing rate after screening and cfDNA testing; PAPP‐A, pregnancy‐associated plasma protein A; PlGF, placental growth factor; SDR, detection rate of screening at the given risk cut‐off.

T21, serum = free Beta hCG, PAPP‐A, AFP, PlGF, DIA; T13/1, Serum = free Beta HCG, PAPP‐A, PlGF. The associated false positive rates for cfDNA testing alone for trisomy 21 and trisomy 18/13 were 4.16% and 4.26%, respectively, while the associated detection rates were 99.232% and 95.176%, respectively. These figures include a cfDNA no‐call rate of 4% considered as positive screening results and published cfDNA performance rates.^16^

While cell‐free DNA testing provides very high detection rates at low false positive rates because of its expense, it is ill‐suited as a primary screen. As a result, several studies have suggested using a contingent approach. In such an approach, lower risk cut‐offs such as 1/1000 are used, and follow‐up testing is performed using cell‐free DNA testing. A recent publication by Chitty *et al.*,^20^ using such an approach based on data from the United Kingdom National Health Service, found that over 80% of patients with risks between 1/150 and 1/1000 chose to undergo NIPT as a follow‐up to conventional screening and concluded that such an approach can be implemented into clinical use.

The current study shows that using a lower risk cut‐off such as 1/1000, we could achieve detection rates of 98% for trisomy 21 and 92.3% for trisomy 18/13 with invasive testing rate of 0.11% and 0.05% (Table 5). Use of a lower risk cut‐off may be difficult to implement because it represents a change in the paradigm of conventional screening as it has existed for decades. However, even using existing cut‐offs, our data show that a contingent protocol can detect 98% of trisomy 21 and 90.5% of trisomy 18/13 with invasive testing rates of less than 0.1%.

Historically, the focus of conventional aneuploidy screening has been on trisomy 21 more than for trisomy 18/13. However, conventional aneuploidy screening performs extremely well in identifying trisomy 18/13 cases, and the data presented here indicate that improved performance can be achieved with the addition of PlGF. Similarly, with cell‐free DNA testing, the focus has been on trisomy 21 rather than trisomy 18 and 13. While cell‐free DNA testing works well in identifying trisomy 18 and 13, the performance is not as good as with trisomy 21. Indeed, detection efficiency of trisomy 18/13 with cell‐free DNA testing may not be as good as conventional screening. For example, in the NEXT trial,^40^ cell‐free DNA detected two fewer cases of trisomy 18/13 than conventional screening once no call results were taken into account. In addition, first trimester screening has been shown to detect a number of other chromosomal abnormalities not directly targeted by the test whereas cell‐free DNA is focused specifically on the disorders being screen for at the exclusion of other chromosomal abnormalities.^41^

The strengths of the current study are that the analytes were tested using assays already approved for clinical use. In addition, we incorporated ultrasound parameters nuchal translucency and nasal bone, which are widely used in the United States. The weaknesses of the study are that it was retrospective and relied on modeling of Gaussian distributions. Although such an approach may be subject to bias towards better screening performance, such an approach has been used widely in this field.^11^, ^42^ Another weakness is that we calculated an overall median MoM value in the trisomy 21 cases instead of using a regression model because most cases were at 12 weeks gestation. It is likely that in future studies, refinements to the parameters will give more precise assessments of false positive and detection rates at individual gestational ages.

A further advantage of the expanded screen is that some of the analytes used in the screen have been reported to be effective in screening for early onset preeclampsia.^22^, ^23^, ^24^ As a result, clinicians and patients ordering an expanded screen could simultaneously be provided with risk assessment for trisomies 21, 18 and 13 as well as early‐onset preeclampsia. Such an approach could speed the implementation of preeclampsia risk assessment as it does not require any additional logistical efforts by the clinicians nor separate billing assessment.

**WHAT'S ALREADY KNOWN ABOUT THIS TOPIC?**

- Conventional Down syndrome screening with ultrasound markers and free Beta hCG and PAPP‐A has been successfully utilized for nearly 20 years to screen for trisomies 21, 18 and 13.
- Cell‐free DNA screening has much higher detection and lower false positive rates but is expensive.

**WHAT DOES THIS STUDY ADD?**

- An expanded conventional screen with nuchal translucency and nasal bone that includes additional serum markers AFP, placental growth factor and dimeric inhibin A can detect 98% of trisomy 21 and 95% of trisomy 18/13 cases at a false positive rate of 1.2% and 0.5%, respectively.
- Offering cell‐free DNA testing to those patients found at increased risk with the expanded screen maintains the detection efficiency but brings the invasive testing rate to an exceedingly low level.
